# Perspectives on RNA Vaccine Candidates for COVID-19

**DOI:** 10.3389/fmolb.2021.635245

**Published:** 2021-03-25

**Authors:** Pobitra Borah, Pran Kishore Deb, Nizar A. Al-Shar’i, Lina A. Dahabiyeh, Katharigatta N. Venugopala, Vinayak Singh, Pottathil Shinu, Snawar Hussain, Satyendra Deka, Balakumar Chandrasekaran, Da’san M. M. Jaradat

**Affiliations:** ^1^ School of Pharmacy, Graphic Era Hill University, Dehradun, India; ^2^ Pratiksha Institute of Pharmaceutical Sciences, Assam, India; ^3^ Department of Pharmaceutical Sciences, Faculty of Pharmacy, Philadelphia University, Amman, Jordan; ^4^ Department of Medicinal Chemistry and Pharmacognosy, Faculty of Pharmacy, Jordan University of Science and Technology, Irbid, Jordan; ^5^ Department of Pharmaceutical Sciences, School of Pharmacy, The University of Jordan, Amman, Jordan; ^6^ Department of Pharmaceutical Sciences, College of Clinical Pharmacy, King Faisal University, Al-Ahsa, Saudi Arabia; ^7^ Department of Biotechnology and Food Technology, Durban University of Technology, Durban, South Africa; ^8^ Drug Discovery and Development Centre (H3D), University of Cape Town, Rondebosch, South Africa; ^9^ South African Medical Research Council Drug Discovery and Development Research Unit, Department of Chemistry and Institute of Infectious Disease and Molecular Medicine, University of Cape Town, Rondebosch, South Africa; ^10^ Department of Biomedical Sciences, College of Clinical Pharmacy, King Faisal University, Al-Ahsa, Saudi Arabia; ^11^ Department of Chemistry, Faculty of Science, Al-Balqa Applied University, Al-Salt, Jordan

**Keywords:** COVID-19, SARS-CoV-2, mRNA, self-amplifying RNA, vaccine, conventional RNA, replicons, mRNA-1273

## Abstract

With the current outbreak caused by SARS-CoV-2, vaccination is acclaimed as a public health care priority. Rapid genetic sequencing of SARS-CoV-2 has triggered the scientific community to search for effective vaccines. Collaborative approaches from research institutes and biotech companies have acknowledged the use of viral proteins as potential vaccine candidates against COVID-19. Nucleic acid (DNA or RNA) vaccines are considered the next generation vaccines as they can be rapidly designed to encode any desirable viral sequence including the highly conserved antigen sequences. RNA vaccines being less prone to host genome integration (cons of DNA vaccines) and anti-vector immunity (a compromising factor of viral vectors) offer great potential as front-runners for universal COVID-19 vaccine. The proof of concept for RNA-based vaccines has already been proven in humans, and the prospects for commercialization are very encouraging as well. With the emergence of COVID-19, mRNA-1273, an mRNA vaccine developed by Moderna, Inc. was the first to enter human trials, with the first volunteer receiving the dose within 10 weeks after SARS-CoV-2 genetic sequencing. The recent interest in mRNA vaccines has been fueled by the state of the art technologies that enhance mRNA stability and improve vaccine delivery. Interestingly, as per the “Draft landscape of COVID-19 candidate vaccines” published by the World Health Organization (WHO) on December 29, 2020, seven potential RNA based COVID-19 vaccines are in different stages of clinical trials; of them, two candidates already received emergency use authorization, and another 22 potential candidates are undergoing pre-clinical investigations. This review will shed light on the rationality of RNA as a platform for vaccine development against COVID-19, highlighting the possible pros and cons, lessons learned from the past, and the future prospects.

## Introduction

Vaccination has had an enormous global impact on the prevention of morbidity and mortality associated with infectious diseases, since the discovery of the smallpox vaccine by Edward Jenner in the 18th century (Jenner, 1800). Immunization with vaccines has led to the eradication of many infectious pathogens, including smallpox, and nowadays, more than 30 diseases can be successfully controlled or prevented through vaccination (Younger et al., 2016; Standaert and Rappuoli, 2017). Over the years, the field of vaccinology has evolved from the traditional whole-cell vaccines, such as live-attenuated and inactivated type, to a modern rational vaccine design based on immunological techniques, genetic engineering, and structural biology (Maruggi et al., 2019). Consequently, several vaccine platforms have been designed, developed, and evaluated to obtain robust immunogenic response and to obviate the safety concerns associated with traditional vaccines. Despite considerable advancement in vaccinology, the emergence of novel pathogens characterized by unpredictable nature, high morbidity, and rapid spreading ability, as the ongoing Coronavirus Disease-2019 (COVID-19) pandemic caused by the Severe Acute Respiratory Syndrome Coronavirus 2 (SARS-CoV-2), had significantly increased the demands for expedited vaccine development as a rapid response to the outbreak.

The COVID-19 outbreak continues to wreak havoc since the first reporting of the disease clusters by the China Office of the World Health Organization (WHO). Later, on January 30, 2020, the WHO declared this outbreak a Public Health Emergency of International Concern (WHO, 2020b). The pandemic caused uncertainty about when or whether the COVID-19 will come to an end due to the dearth of risk assessment, rapid infectious ability (R_0_ of 2–3), and the predisposition of severe disease complications in aged patients and comorbid conditions (Guo et al., 2020). Given the incubation period of 5–6 days (maximum of 14 days) (WHO, 2020a), the clinical manifestations might vary from signs and symptoms related to upper respiratory tract infections like sore throat and rhinorrhea to mild-moderate clinical signs such as cough, fever, difficulty in breathing, myalgia, and confirmed pulmonary infiltrative lesions (Kotta et al., 2020a; Shinu et al., 2020) to severe neurologic symptoms like encephalopathies. Laboratory data have demonstrated a lower erythrocyte count, hematocrit volume, hemoglobin level, lymphocyte count, leukocyte count, and albumin level, but an increase in aspartate aminotransferase, alanine aminotransferase, and C-reactive protein (CRP) levels in the mild COVID-19 cases (Jin et al., 2020). Intensive care unit (ICU) patients were found to exhibit elevated neutrophil and leukocyte count, higher creatine, creatine kinase, and D-dimer levels (Wang et al., 2020a). Elevated early pro-inflammatory cytokines like interleukins (IL2, IL6, and IL10) and Tumor necrosis factor (TNF) in ICU subjects reflect the cytokine storm leading to acute respiratory distress syndrome (ARDS) and progressive multiple organ failure (Huang et al., 2020). As reported by retrospective cohort studies, a higher prevalence of COVID-19 was observed among the aged patients with co-morbidity, and greater quick sequential organ failure assessment (qSOFA) score and D-dimer level (>1 μg/ml) (Guan et al., 2020; Huang et al., 2020). Other risk factors such as sex, smoking habits, and blood group have also been reported to be linked to the prevalence of the disease (Li H. et al., 2020). The fact that continuous health service delivery was highly affected due to considerable morbidity and mortality rates, and psychological impacts among healthcare workers (Casigliani et al., 2020; Kursumovic et al., 2020), further stressed the need for an effective COVID-19 vaccine. Successful vaccination will not only protect the subject but also lead to the immunization of a large population and the development of herd immunity against the virus to curtail the viral spread promptly.

Immediately after the first publication of the SARS-CoV-2 viral genome, the race for the development of potent and safe vaccines has seen an unprecedented and unimaginable scale and pace—referred to as “pandemic pace.” Currently, around 232 potential vaccine candidates are in various phases of development (WHO, 2020c). Diverse vaccine platforms have been exploited to end up with a handful of successful vaccines at a pandemic pace after the high attrition in developmental stages. Although traditional vaccines, such as live-attenuated or inactivated pathogens and subunit vaccines may provide long-term protection against SARS-CoV-2, the pre-requisites for rapid design, development, and large-scale production are difficult to encounter through these approaches. Similarly, peptide-based platforms conferring lower immunogenicity is another concern (Li et al., 2014). Therefore, the ribonucleic acid (RNA) platform has witnessed huge interest because of its desired safety profile, higher efficacy, lower production cost, and rapid development time (Ahammad and Lira, 2020). Indeed, RNA-based vaccines are being anticipated as one of the rapid solutions for the pandemic crisis due to their versatile nature, simple manufacturing process, and the pre-requisite of only pathogenic sequence for vaccine development. Additionally, advanced self-amplifying and trans-amplifying RNA vaccine candidates allow potent and durable antigen production *in vivo* in lower doses because of their inherent immuno-stimulatory properties (de Queiroz et al., 2020). The recent interest in the development of messenger RNA (mRNA) vaccines has been fueled by the state of the art technologies that enhance mRNA stability and improve vaccine delivery. Interestingly, owing to its rapid designing and manufacturing ability, the initial phase I human trial of a novel lipid-derived nanoparticle (LNP)-formulated mRNA vaccine, mRNA-1273, that encodes for the pre-fusion stabilized spike (S) protein of SARS-CoV-2, was initiated in the United States (US) by the developer Moderna, Inc. and the National Institute of Allergy and Infectious Diseases (NIAID) (Moderna, Inc., 2020b) within 10 weeks after sequencing of SARS-CoV-2 genome. The success of the preliminary study of mRNA-1273 in phase III trials (NCT04470427) paved the way for many other developers to design RNA-based vaccines as a prophylactic options against COVID-19. In light of the recent advancements and ongoing progress, this review will discuss the rationality of the RNA platform for potential vaccine development against COVID-19, highlighting the possible pros and cons, lessons learned from the past, and future prospects.

## Genomic Organization of SARS-CoV-2 and Host Immune Responses

After the reporting of mysterious pneumonia cases from Wuhan in China, the complete genome of the causative organism was obtained with the aid of next-generation sequencing techniques and made available on the virological website (can be accessed at http://virological.org) on January 11, 2020 (Zhang and Holmes, 2020). Subsequent multiple-genome sequencing and phylogenetic analyses revealed 79.5% similarity with the severe acute respiratory syndrome coronavirus (SARS-CoV) and confirmed that the novel coronavirus i.e. SARS-CoV-2 belongs to the genus Betacoronavirus (Wu et al., 2020). Later, it was reported that SARS-CoV-2 exhibits the highest (96.2%) sequence identity to Bat CoV RaTG13 at the nucleic acid level (Zhang T. et al., 2020). Nonetheless, the genomic features of SARS-CoV-2 significantly differ from Bat CoV RaTG13, which suggests that the sequence identity implies toward the possible natural host for SARS-CoV-2 as the bats. Moreover, the intermediate host for the human transmission is suspected to be the Malayan pangolins, which showed higher resemblance in amino acid sequence as well as similar mutations in the receptor-binding activity via the receptor binding-domain (RBD) of the spike protein of SARS-CoV-2 (Zhang T. et al., 2020; Zhang and Holmes, 2020).

**FIGURE 1:**
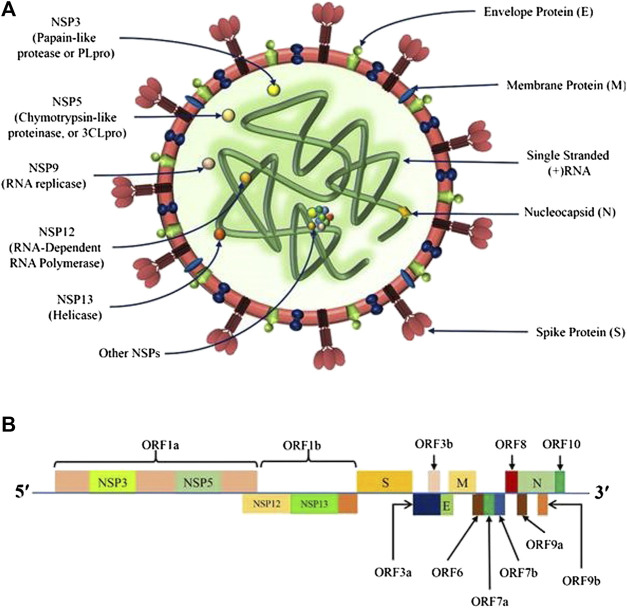
Genomic organization of SARS-CoV-2; **(A)** The structure of SARS-CoV-2 containing spherical crown-like lipid envelope showing structural proteins, namely Spike (S), Envelope (E), Nucleocapsid (N), and Membrane (M) proteins surrounding the non-segmented (+)-sense single stranded RNA encoding for several non-structural proteins (NSPs); **(B)** The SARS-CoV-2 genome representing the arrangement of - 5′ untranslated region (5′-UTR) caps, ORF1ab (replicase), S, E, M, N, and other genes encoding for the NSPs like ORF 3, 6, 7a, 7b, 8, and 9b; and 3′-UTR (poly-A tails).

Coronaviruses, including SARS-CoV-2, belong to the Coronaviridae family containing spherical crown-like lipid envelope surrounding non-segmented positive-sense single-stranded RNAs (Figure 1A) (Hasöksüz et al., 2020; Lundstrom, 2020). The SARS-CoV-2 genome exhibits the following order of arrangement-5′ untranslated region (UTR) caps; open reading frames 1ab or ORF1ab (replicase); Spike (S) gene (S1/S2); Envelope (E) gene; Membrane (M) gene; Nucleocapsid (N) gene; other genes encoding for accessory proteins like ORF 3, 6, 7a, 7b, 8, and 9b; and the 3′-UTR (poly-A tails) (See Figure 1B) (Malik, 2020). The genome encoded structural proteins viz. S, E, M, and N proteins, which are necessary for the assembly of structurally complete virions during the virus replication cycle. The Spike (S) glycoproteins, existing in trimeric forms, are membrane fusion proteins with distinct functions, i.e. the amino-terminal or S1 subunit is involved in the RBD, whereas the carboxy-terminal or S2 subunit is responsible for the formation of the stalk, and thereby, assists in virus fusion. In general, the S protein recognizes the host angiotensin-converting enzyme (ACE) receptor, and leads to membrane fusion and cellular entry (Kotta et al., 2020b), while E protein mediates the virion assembly and their release, and M protein describes the envelope shape (Alanagreh et al., 2020; Malik, 2020). On the other hand, N proteins are associated with the RNA genome packaging during viral replication to form the complete virions (Alanagreh et al., 2020). Several other accessory proteins are also encoded by the genome of SARS-CoV-2 with overlapping compensatory functions (Figure 1). The details of the replication cycle and pathogenesis of the SARS-CoV-2 virus can be reviewed in our recent publication (Borah et al., 2020).

Following infection, SARS-CoV-2 elicits an innate immune response on recognition of viral pattern-associated molecular patterns (PAMPs) by the pattern-recognition receptors (PRRs) (Tay et al., 2020). The viral double-stranded RNA (dsRNA) is the most relevant PAMP in the case of SARS-CoV-2, which can be sensed by Toll-like receptors (TLRs), retinoic acid-inducible gene 1 (RIG-1), and melanoma differentiation-associated protein 5 (MDA5). In the lungs, the local tissue injury also liberates damage-associated molecular patterns (DAMP_S_), which further contribute to the inflammatory conditions. The subsequent inflammatory response imparts immediate immunity through the activation of interferon (IFN) pathways by up-regulating the production of pro-inflammatory cytokines like interleukin 6 (IL-6), which results in neutrophil recruitment and initiate SARS-CoV-2-specific adaptive responses (Channappanavar et al., 2014). It is evident that the innate immune response toward SARS-CoV-2 infection is aberrant (Blanco-Melo et al., 2020). For instance, the primary interferon-mediated responses are seemed to be inhibited by SARS-CoV-2 in the initial stages of the infection, and therefore, fail to limit the viral replication. Moreover, in severe COVID-19 cases, uncontrolled local inflammation or so-called “cytokine storm” leads to inflammatory tissue damage, and ARDS (Borah et al., 2020; Wang et al., 2020a). Severe COVID-19 cases also revealed higher levels of IL-2, IL-6, IL-10, and TNFα (Wang et al., 2020b; Chen et al., 2020). Those ICU admitted COVID-19 patients exhibited increased plasma levels of IL-2, IL-7, IL-10, granulocyte colony-stimulating factor, monocyte chemoattractant protein- 1, interferon-inducible protein 10, and TNF-α than the non-ICU admitted patients (Huang et al., 2020). The up-regulation of chemokines further leads to the recruitment of inflammatory cells, including macrophages, neutrophils, and natural killer cells resulting in immunopathological disorders (Blanco-Melo et al., 2020).

It is believed that the production of neutralizing antibodies against SARS-CoV-2 is vital for the development of a successful human vaccine (Shang et al., 2020). Most of the current vaccine technologies are focusing on the production of neutralizing antibodies against S protein responsible for an attachment to the ACE2 receptor (Salvatori et al., 2020). Notably, the hidden RBD domain in S protein is the most predominant state, which is used by the virus to evade immune responses, and interferes with the functioning of RBD-based mRNA vaccines (Shang et al., 2020; Zhang and Kutateladze, 2020). Considering this, researchers are aiming to elicit neutralizing antibodies toward the less immunogenic S2 subunit (Shang et al., 2020) or other antigens such as N protein. Remarkably, it is evident that memory B cells produced in COVID-19 patients can also provoke a rapid recall (memory-based) response on subsequent exposure to SARS-CoV-2 (Brouwer et al., 2020).

The antigen-presenting cells (APCs) present the SARS-CoV-2 epitopes via human leukocyte antigen (HLA) class I, which differentiates the naive CD8^+^ cells to cytolytic effectors to destroy the virus-infected cells. Similarly, activation of T helper 1 (Th1) cells through HLA class II presentation further augments the CD8^+^ cell-mediated response. On the other hand, HLA class II restricted Th2 and follicular helper cells trigger the production of virus specific antibodies. Thus, effective viral clearance involves a combined effect of CD8^+^ and CD4^+^ cell-mediated B-cell and T-cell responses (Channappanavar et al., 2014). Biopsy of COVID-19 positive subjects also revealed a higher level of pro-inflammatory Th17 (Kadkhoda, 2020). Evidently, SARS-CoV and Middle East Respiratory Syndrome (MERS)-CoV infections demonstrated the induction of long-term persistence of memory T-cells after infection, lasting for ∼6 years and ∼2 years, respectively (Tang et al., 2011; Zhao et al., 2017), indicating the potential for long-lasting protective immunity in SARS-CoV-2 infection. Undoubtedly, the robust memory T-cell formation supports the ultimate purpose of an effective vaccine. However, taking into account the complex T cell-mediated immunity, a biphasic response model has been described earlier for SARS-CoV (Chen et al., 2010), which suggests an early CD8^+^ cell response for viral clearance followed by the T-cell exhaustion as a result of prolonged viral persistence in some patients (Flanagan et al., 2020). This biphasic model explains the poor outcomes observed in the elderly subjects with reduced T-cell pool, and the superior effects in subjects (like children) with diverse naive T-cell repertoires (Flanagan et al., 2020; Vardhana and Wolchok, 2020). Therefore, the potential role of CD4^+^ and CD8^+^ T-cells in controlling the virus highlighted the necessity to explore T-cell induction in vaccine development approaches against SARS-CoV-2.

## RNA as a Vaccine Platform: Proof of Concept and Beyond

The RNA molecules encode the genetic information necessary for protein synthesis in all livings. Logically, the expression of a defined set of proteins to immunize a vaccinated subject would be possible through the inoculation of RNA molecules. The proof of concept was provided three decades ago, when mRNA injection resulted in the expression of an encoded protein (Wolff et al., 1990), and induced desirable immunogenic responses in a murine model (Martinon et al., 1993). Mass basis direct inoculation of similar doses of plasmid DNA and mRNA (in a sucrose formulation) exhibited the expression of reporter genes with equivalent efficiency (Wolff et al., 1990). These findings paved the way for developing more solid evidence on the elicitation of immunogenic responses by the RNA vaccines. Several experiments have demonstrated the capability of RNA molecules in expressing different proteins *in vivo* through various gene targets like reporter genes (Hoerr et al., 2000), allergens (Bachmann and Kündig, 2017), viral antigens (Scorza and Pardi, 2018), and tumor antigens (Granstein et al., 2000). Notably, both antibody-mediated and T-cell (CD4^+^ and CD8^+^) mediated immune responses along with functional immunity were evident from these studies. Nevertheless, until recently, the nucleic acid-based immunization has been dominated by the experimental plasmid DNA vaccines, as plasmids have been regarded as the highly stable form of nucleic acid, and are comparatively faster and easier to produce. Although the initial success of gene-based immunization, particularly with DNA in small animals, has generated a high level of expectations, insufficient efficacy in higher animals as well as in humans led to the waning of the enthusiasm (Weiner and Nabel, 2018). To optimize the DNA vaccines, tremendous efforts have been made through improved DNA construction, integration of immuno-stimulatory agents and incorporation of advanced delivery approaches like electroporation (Chiarella et al., 2013) but till now no DNA vaccines for human use have been approved by the regulatory authorities. Interestingly, in the past three decades of development, it has been realized that the RNA molecules not only can serve as the genetic carrier of protein translation but can also control the enzymatic as well as regulatory functions by interacting with a myriad of host factors. Therefore, the RNA-based vaccine platform offers several advantages over the DNA one. Unlike DNA, the RNA molecules do not require entry into the nucleus through an additional barrier, i.e. nuclear membrane for transcription; rather it is directly available after the inoculation into the cytoplasm for translation of the encoded proteins (Liu, 2019). The presence of an intact nuclear membrane in case of non-dividing cells like myocytes is another problem in immunization with DNA vaccine. For instance, cytoplasmic microinjection of plasmid DNA into non-dividing cells demonstrated a low level of protein expression when compared to direct inoculation into the nucleus (Zabner et al., 1995). Moreover, the potential issue of integration of the foreign DNA with the host genome is obviated by the RNA. Of importance, the host immune response factors are essential for counteracting the RNA viruses following their detection (Jensen and Thomsen, 2012). Therefore, certain RNA molecules may initiate the triggering of innate immune responses, which subsequently lead to a more efficient and strong adaptive response. For example, exogenous dsRNA is recognized as a signaling molecule capable of eliciting immune responses in the host (Kaldis et al., 2018). Likewise, a fully optimized RNA-based vaccine confers an advantage over the DNA vaccine, as it induces the innate immune response by utilizing various cellular pathways in response to foreign RNA (Elion and Cook, 2018), including endosomal receptors like TLR3, TLR7, and TLR8, and cytoplasmic receptors like MDA-5, NLRP3, RIG-I, and NOD2 (Lazzaro et al., 2015; Chen et al., 2017; Rauch et al., 2018). Immunization with both DNA and RNA vaccines may lead to the upregulation of cytokine expressions, including chemokines (CXCL9, CXCL10, and CXCL11) and type I interferons that recruit immune cells like dendritic cells (DCs) and macrophages, followed by enhancement of the adaptive immune responses (de Queiroz et al., 2020). Additionally, RNA-based vaccines also demonstrated an intrinsic adjuvant effect. However, the relative stability of RNA-based vaccines is considered a major concern.

Typically, inoculation of RNA can be achieved through various routes such as intramuscular (Erasmus et al., 2020), subcutaneous (Martinon et al., 1993), intravenous (Mockey et al., 2007; Broos et al., 2016), intradermal (Pardi et al., 2017; Golombek et al., 2018), intranodal (Bol et al., 2015), and intrasplenic (Broos et al., 2016), and by gene gun method (Qiu et al., 1996; Aravindaram and Yang, 2009); therefore, exhibiting versatility in immunization against infectious as well as non-infectious diseases. Several other strategies have been considered for improved RNA vaccine delivery including, microinjections (Golombek et al., 2018), protamine condensation (Zhang et al., 2018), RNA patches (Koh et al., 2018), RNA adjuvants (Schlake et al., 2012), encapsulation of RNA in lipids and/or polymer-based nanoparticles (Mukherjee et al., 2019), and *in vitro* transcribed (IVT) mRNA mixing with a complexing agent (Sahin et al., 2014). Even though less is known on the mechanism, it is hypothesized that RNA is immediately exposed to the tissue RNAases following its administration (Probst et al., 2006), which limits the cellular uptake of the functional RNA mediated by the membrane domains abundant with lipid rafts and caveolae, and scavenger receptors. Additionally, the 2ʹ-hydroxyl group of the ribose moiety confers a non-stable double helix due to steric hindrance leading to the hydrolysis of mRNA. Cytoplasmic accumulation of RNA following cellular internalization initiates the protein translation, mimicking the pathogenic infection, and tumor antigen expression to induce T-cell mediated immune responses like DNA and viral vectors (Probst et al., 2006). Apart from being a potent stimulator of the innate immune response, RNA vaccine may also potentiate the B-cell mediated immunity and antigen-directed antibody production. It is suggested that RNA vaccine initially leads to the local antigen expression to promote the major histocompatibility complex (MHC) presentation followed by the antigen-directed immune responses through stimulation of the innate responses. Moreover, RNA vaccination seems to mimic an acute infection in terms of antigen-specific rapid immune responses that tend to subside quickly (Probst et al., 2006).

## Types of RNA Vaccines and State of the Art Technologies

**FIGURE 2:**
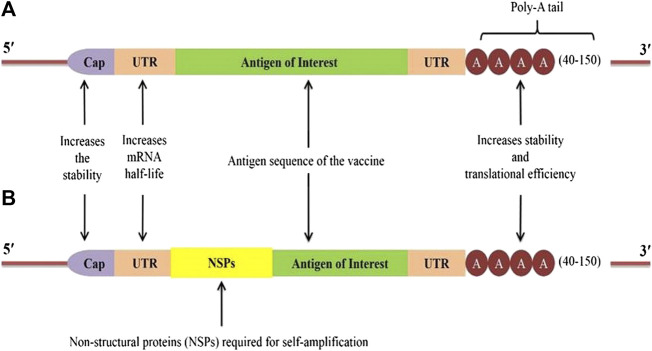
Construct of two types of RNA vaccines: **(A)** A typical conventional mRNA construct with Cap, untranslated regions (UTRs), antigen of interest, and **poly-A tail**; **(B)** self-amplifying mRNA or Replicons construct with the sequences of non-structural proteins (NSPs) derived from another virus (e.g. Alpha virus) introduced between the 5ʹ-UTR and the antigen of interest.

Generally, two major types of RNA vaccines are available: 1) Conventional or non-amplifying type mRNA, and 2) RNA replicons engineered from viruses with positive-stranded RNA (Figure 2). These RNA vaccines are discussed below.

### Conventional mRNA Vaccine

**FIGURE 3:**
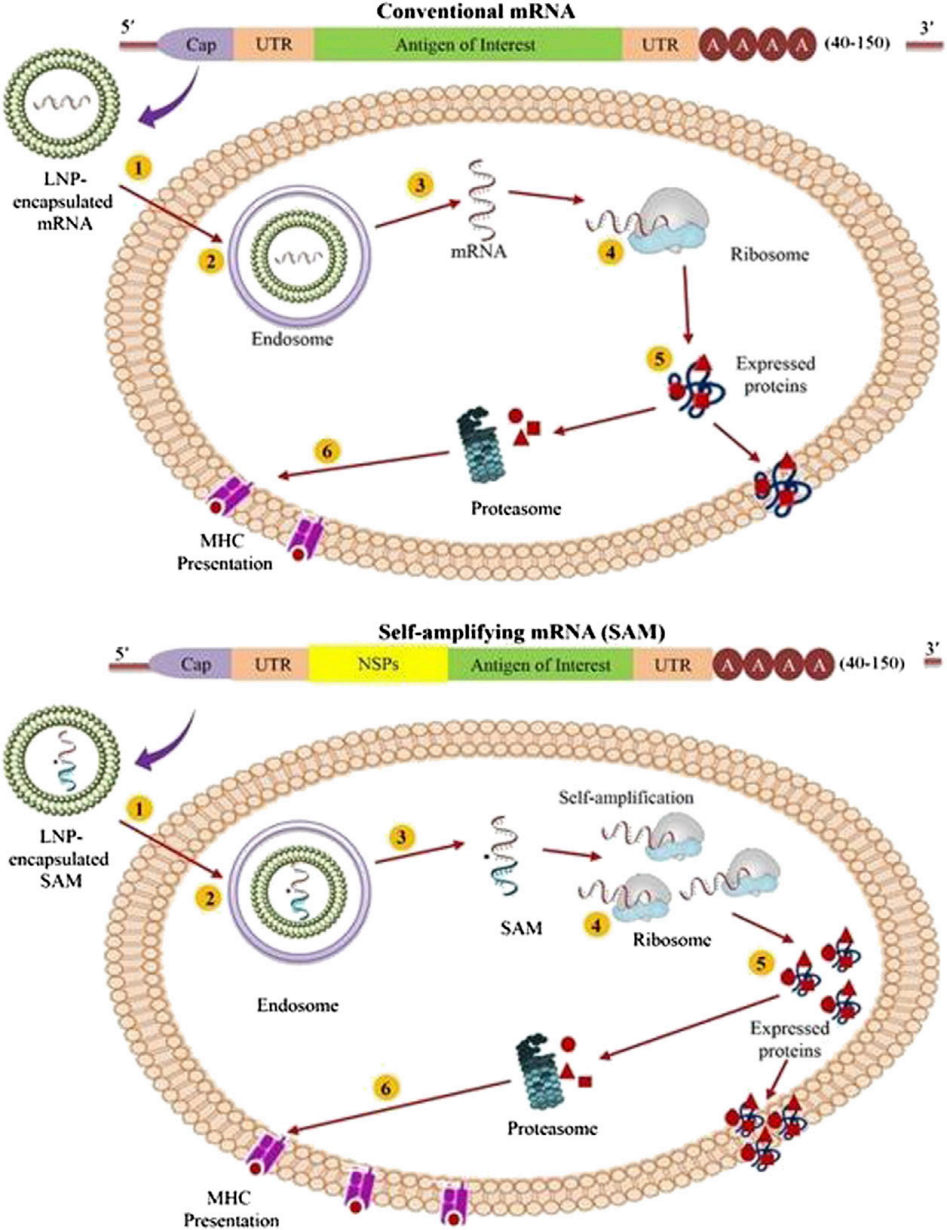
Diagrammatic representation of mechanism of antigen expression by the conventional mRNA **(Top)** and self-amplifying mRNA (SAM) vaccines **(Bottom)**. (1) In this illustration, both conventional mRNA and SAM are shown to be formulated in lipid-derived nanoparticles (LNPs) to provide better stability; (2) The LNP formulated mRNA enters the cell through membrane-derived endocytosis processes; (3) The mRNA content shows endosomal escape to reach the cytosol; (4) In case of conventional type, the escaped mRNAs are immediately translated by the ribosomes to generate the protein of interest **(Top)**, while SAM constructs undergoes translation to produce the replicase complex to exhibit self-amplification of the encoded mRNA, followed by translation of the antigen of interest to express the desired protein **(Bottom)**; (5) Then the expressed proteins undergoes subsequent post-translational modification to appear as trans-membrane, intracellular or secreted protein; and (6) The expressed proteins are then broken down to peptides by the proteasome, and the peptide formed are presented to the immune system by the major histocompatibility complex (MHC).

Owing to their distinct advantages and disadvantages, mRNA vaccines are the simplest type of RNA vaccine as they consist of only a relatively smaller size of RNA molecule of interest (Figure 2A). The development of mRNA vaccine involves identification of antigen of choice followed by gene sequencing, synthesis, cloning into DNA template plasmid, and *in vitro* transcription. Once mRNA enters a cell, it utilizes the host cell machinery to translate mRNA *in vivo* to produce the corresponding antigen, and the final cellular location of the protein synthesis can be either intrinsic to the natural gene sequences or engineered to guide the gene to the desired site (Figure 3) (Maruggi et al., 2017). Moreover, the absence of any accessory proteins excludes the probability of unwarranted immune responses within the host (Schlake et al., 2012). However, the functionality of the conventional mRNA is limited under *in vivo* conditions as cells restrict the duration of mRNA expression, thus, requiring a higher dose (Ross, 1995). Considering the fully synthetic nature, it is virtually possible to design any gene sequence *in silico* that can later be synthesized and delivered in the vaccine form to test the *in vivo* immunogenicity in experimental models. Furthermore, the development of mRNA vaccines against Zika viruses has demonstrated that desirable antigen sequences can be designed and rapidly evaluated to produce vaccines with minimal codon usage, improvised leader sequences, efficient neutralization capacity, and/or minimal cross-reactivity (Richner et al., 2017). In the recent past, significant efforts have been made to optimize RNA stability and improve the delivery of RNA vaccines (Figure 2). These methods include the addition of 5ʹ-cap and Kozak sequences, 3ʹ-poly-A sequences, and chemical alterations of RNA using nucleotide derivatives, e.g. pseudouridine, which markedly augments the *in vivo* protein expression (Karikó et al., 2008; Warren et al., 2010). The modification in nucleoside bases coupled with chromatographic techniques has been utilized to produce the modified mRNA with superior translation capacity and devoid of contaminants such as dsRNA, short RNA stem-loops, and intermediates of RNA replication (Weissman et al., 2013). Notably, several researchers have experimented mainly with the naked mRNA, formulated in a buffer solution to elicit the immune responses but are highly susceptible to the degradative enzymes. Therefore, the use of advanced formulation techniques, such as lipid and LNP encapsulation (Zeng et al., 2020), exosome encapsulated RNA (Hood, 2016), RNA-transfected DCs (Benteyn et al., 2015), and continuous-flow microfluidic devices enabled the desirable production of nanoparticles (Jahn et al., 2008; Valencia et al., 2012). Likewise, the RNActive technology aims to enhance the adjuvant properties of the formulation, which contains an unmodified, naked, and codon-optimized mRNA. The potency of this formulation mainly depends on the carrier comprising protamine amalgamated non-coding RNA, which can activate the TLR7 (Alberer et al., 2017; Edwards et al., 2017). It is also reported that coupling of 1-methylpseudouridine-modified naked mRNA to TLR2 and TLR7 agonists with ovalbumin as antigen also increases the immune responses in mice model (Loomis et al., 2018). The codon optimization also demonstrated robust immunogenicity and antigen expression. The mRNA enriched in guanine and cytosine content with optimized untranslated regions (UTRs) is shown to be superior to a nucleoside-modified counterpart, both *in vitro* and *in vivo*.

Likewise, codon-optimized mRNA encapsulated with LNPs provoked robust immunogenicity against influenza and rabies antigens in NHPs (Lutz et al., 2017). On the contrary, intradermal inoculation of 1-methylpseudouridine-modified mRNA with LNPs yielded about 20 folds higher protein expression as compared to the nucleoside-unmodified but codon-optimized counterpart. However, discrepancies among these observations are yet to be defined but these findings highlighted that both techniques are superior to the unmodified mRNA. Codon-optimized as well as nucleoside-modified mRNA encapsulated with LNPs can elicit a robust innate immune response and infiltration of neutrophils, DCs, and monocytes to the inoculation site, along with the upregulation of CD80^+^ receptors, CD86^+^ receptors, and IFN-inducible genes e.g. CXCL10 and Mx1 (Liang et al., 2017; Lutz et al., 2017). Apart from these, factors like the production of thermostable RNA molecules, detection of RNA by the immune cell receptors, and selection of proper route of administration to decrease the RNA degradation all influence the RNA vaccination (Lundstrom, 2018; Uchida et al., 2018; Zhang et al., 2019).

### Self-Amplifying mRNA Vaccine

Self-amplifying mRNA (also called Replicons) vaccines are based on the engineered RNA molecules (∼10 kb) that encode for the replicative factors required for RNA amplification within the host cell (Figure 2B). These replicons are considerably more potent than the conventional mRNA vaccines. Usually, the engineered RNA molecules are obtained from the single-stranded positive RNA viruses, like alphaviruses, flaviviruses, and picornaviruses (Lundstrom, 2016; Tews and Meyers, 2017). Among these, the most explored one is the alphavirus genomes including, Semliki Forest, Sindbis, and Venezuelan equine encephalitis viruses (Atkins et al., 2008; Ljungberg and Liljeström, 2015). The replicons are produced by replacing the genes encoding for structural proteins of the virus with the desired antigens, which are capable of self-amplifying within a target host cell using the RNA polymerase encoded in the replicon (Figure 3). As these replicons do not possess genes required for structural protein expression, it is unlikely to produce virions capable of infecting other cells. Instead, a high level of the desired antigens is expressed from the sub-genomic RNA. However, these RNA replicons readily form virus-like particles when the viral structural proteins are provided in cell culture in *trans* or as synthetically derived RNA (Maruggi et al., 2019). Moreover, it is also possible to formulate the replicons synthetically rather than using any virus materials (Geall et al., 2012). Generally, delivery of the replicons can be achieved in the form of virus-like particles, plasmid DNA, and IVT RNA, which are capable of substantial induction of stronger immune responses than mRNA (Fuller and Berglund, 2020). The potent immunogenicity and the ability to deliver these replicons in synthetic formulations to a high scale make the self-amplifying RNA vaccine an attractive approach. The DNA plasmid-based replicons combine the greater stability of the DNA product with higher levels of antigen expression by the replicons to induce stronger immune responses than the conventional DNA-based vaccines (Berglund et al., 1998). On the other hand, a strategy comprising two RNA vectors, one containing the gene encoding for replicase and the other encoding for the antigen of interest has also been described (Beissert et al., 2020). The *in trans* availability of replicase provided by one of the RNA mediates replication of the immunogen-encoding RNA. Although a higher amount of genes encoding for replicase were required, the induction of robust antibody-mediated responses was evident in mice after inoculating them with immunogen-encoding for hemagglutinin (influenza protein) at low (nanogram) doses. Importantly, this strategy offers significant advantages, particularly in manufacturability, increased safety, and ease of optimization as compared with conventional replicons. The use of two separate RNAs also avoids the risk of transferring viral glycoproteins to the extracellular vesicles and other host cells. The trans-amplifying mRNA strategy permits the incorporation of shorter RNA for the scaled-up manufacturing process, which is a challenging issue for the conventional longer self-amplifying RNAs. However, the production of two RNAs and the efficient delivery of them into the cell remain a big challenge (Beissert et al., 2020). Considering the self-amplification of this type of vaccine, a high level of antigen expression for an extended period can be achieved with low doses of vaccine (Brito et al., 2014; Leyman et al., 2018). Nevertheless, the stability and manufacturing of the self-amplifying mRNA vaccines are quite challenging compared to the conventional type. The dsRNA intermediates also trigger the innate immune responses conferring an adjuvant effect. Similarly, LNP-encapsulated replicons may act as an adjuvant for the subunit inactivated influenza vaccine to provoke the H1-specific effector CD8^+^ cells, and augment the magnitude of H1-specific CD4^+^ cells (Magini et al., 2016). Notably, in the case of replicons, the role of innate immunity is quite complex. The LNP-encapsulated replicons induce strong pro-inflammatory effects by robust initiation of type I IFN responses at the injection site (Pepini et al., 2017). Although IFN activation is favorable for potent immune responses, early type I IFN response may impair the replicon expression and its potency (Maruggi et al., 2013). Usually, type I IFN determines the differentiation of CD8^+^ cells into cytolytic effectors but it also leads to T-cell exhaustion (De Beuckelaer et al., 2016). It is expected that the stimulation and inhibition of CD8^+^ cell responses to replicons depend on the timing and the intensity of the IFN-mediated responses (Sheehan et al., 2006). Therefore, optimization of self-amplifying mRNA vaccines can be achieved by taking advantage of both intermediates of the viral replication process (such as dsRNA) and host adaptive mechanisms (Geall et al., 2012). Other possible strategies to increase the potency of replicons include codon modification to produce IFN-insensitive mRNA, novel designing to restrict the IFN induction, and addition of small-molecule modulators to control the IFN signaling cascade (Iavarone et al., 2017). Another feature of the replicon-based platform is its ability to encrypt multiple antigens of interest in the same replicon. This approach allows expressing both the target antigen and biological adjuvant to enhance the vaccine efficacy, and thus, can be useful in the development of a single combo vaccine capable of targeting multiple pathogens. Proof-of-concept of multiple antigen delivery with LNP-encapsulated replicons was also reported in the literature for human cytomegalovirus and influenza virus proteins (Brito et al., 2015; Magini et al., 2016). It has been demonstrated that the replicons formulated in 1,2-dioleoyl-3-trimethylammonium-propane (DOTAP) nanoparticles, DOTAP liposomes, and dimethyl-dioctadecylammonium bromide (DDA) liposomes induce the highest protein expression *in vitro*, and DOTAP nanoparticle encapsulated replicons elicit humoral and cellular immune responses *in vivo* (Anderluzzi et al., 2020). Further insights into the plausible mechanisms involved in the interaction of replicons with the host immunity will facilitate the rational design of superior self-amplifying RNA vaccines. For instance, the two RNA vectors approach could allow further improvement using newer strategies in mRNA technology like nucleoside modification, sequence stabilization, and codon optimization, which are not likely to be possible for conventional replicons (Beissert et al., 2020).

### Progress in Clinical Trials

It is worth mentioning that some of the RNA-based personalized cancer vaccines have also been subjected to clinical trials. Apart from the intranodal injection of synthetic RNA vaccines, the RNA lipoplex nanoparticle-based formulations (second generation RNA vaccines) have been undergoing clinical development (Grabbe et al., 2016). For example, a phase I/II trial is underway for the assessment of RNA-lipoplex nanoparticles (lipoMERIT) responsible for the expression of shared tumor-associated antigens (TAA) against triple negative breast cancer (NCT02316457), and advanced melanoma (NCT02410733). The preliminary data suggests a better safety and tolerability profile in about 40 patients. Additionally, a high-level vaccine-induced immunogenicity exhibiting growth of pre-existing protection, and *de novo* elicitation of antigen-specific immunity was reported on multiple administration of lipoMERIT (Lundstrom, 2018). In another clinical trial, intranodal ECI-006 vaccine containing TriMix and five TAAs mRNA have been evaluated for anti-tumoral immune response in melanoma patients (NCT03394937). On the other hand, immunization is in a process to assess the intradermal mRNA vaccine alone or in combination with anti-CD40 co-stimulatory antibody for human papilloma virus-positive head and neck squamous cell carcinoma (NCT03418480). A phase I trial will evaluate the development of anti-tumoral immune response by V941 or mRNA-5671 (an LNP-formulated mRNA vaccine) (NCT03948763). Another clinical trial is designed to assess the effectiveness of the neoantigens-encoded personalized mRNA-based vaccine in patients with non-small-cell lung carcinoma (NSCLC) and advanced esophageal cancer (NCT03908671). Apart from these, several mRNA-based COVID-19 vaccines are also undergoing clinical trials, which will be detailed in *Challenges and Opportunity Gaps in RNA Vaccine Development in Pandemic Situation*.

## Quest for an Effective COVID-19 Vaccine

Although pandemics wane over time, a sporadic wave of clusters may manifest. As the potential threat from the SARS-CoV-2 virus is likely to be continued, the development of an effective vaccine through intense global efforts is the only way to contain the ongoing infections as well as possible sporadic waves. Previous data from MERS-CoV and SARS-CoV pandemic led to a renaissance in vaccine development against SAR-CoV-2, right after the declaration of the outbreak (Prompetchara et al., 2020). The majority of the targeted antigens including S protein or S domain subunits were selected based on previous knowledge (Kandeel et al., 2020; Smith et al., 2020). Based on previous findings, a set of SARS-CoV-2 specific epitopes were screened to accelerate the vaccine development program (Lee and Koohy, 2020; Lucchese, 2020). Moreover, earlier experiences with similar outbreaks have emphasized the necessity of more advanced or newer vaccine platforms capable of adapting to novel emerging diseases, like COVID-19 (Marston et al., 2017). A striking feature of the ongoing COVID-19 vaccine development is the diversity of the platform technologies available, such as nucleic acid (RNA and DNA), protein subunit, virus-like particles, inactivated virus, viral vectors (both replicating and non-replicating), and live attenuated virus. However, most of these platform technologies are not the basis for existing approved vaccines but interesting experimental findings from fields like oncology are alluring the developers to explore the next-generation tools offering rapid and robust development process to meet the demand of the situation (Le et al., 2020). Nucleic acid-based platforms, both DNA and RNA, followed by the recombinant-subunit preparations confer tremendous potential for a rapid generation as they utilize synthetic processes, and are feasible with reverse genetics and next-generation sequencing (Lurie et al., 2020). An ideal vaccine platform would demonstrate progress into the clinical trials within 16 weeks from the viral sequencing exhibiting consistent immune responses and suitability for large-scale production (Lurie et al., 2020). Considering the potential vaccine candidates, the novel platform based on mRNA (also DNA) presents greater flexibility with regards to manipulation of antigens and rapidness in production. The mRNA-1273 developed by Moderna, Inc. entered into the clinical trials within 10 weeks after the SARS-CoV-2 genome sequencing. On the other hand, viral vector-based platform exhibits long-term stability and a higher level of protein expression to induce robust immune responses. Several vaccines based on recombinant proteins are also licensed for other diseases, and many such candidates could capitalize on the large-scale production capacity. However, discussion of the various platform technologies is beyond the scope of this article and is extensively reviewed by Li et al. (Li L. et al., 2020).

## Representative RNA Vaccines for COVID-19 Undergoing Clinical Trials

To curtail the spread of SARS-CoV-2 infection, unprecedented investment has been made in COVID-19 vaccine development and subsequent scale up for large production. Following this, the WHO periodically provides revised and updated details on the current landscape of vaccine candidates against COVID-19 (WHO, 2020c). As of December 29, 2020, there were 172 vaccine candidates under pre-clinical trials, and another 60 vaccines were at least in phase I clinical trials or above. Of the previous vaccines, a total of 7 (12%) that are under clinical trials, and 22 (12.8%) from pre-clinical trial settings are based on the RNA platform technology. As the vaccine development process is continuously and rapidly evolving, the next sub-section will only discuss the key vaccine candidates under clinical development and currently available information as of December 29, 2020 (also refer to Table 1).

**TABLE 1:** Examples on potential RNA-based COVID-19 vaccine candidates currently undergoing clinical trials.

Name of the vaccine	RNA type	Sponsor(s)	No. of doses	Dosing interval (days)	Clinical trial phase and identifier
mRNA-1273	LNP-encapsulated mRNA	Moderna inc. and NIAID	2	0, 28	Phase III NCT04470427
					Phase II NCT04405076
					Phase I NCT04283461
BNT162 (1a, b1, b2, c1)	LNP-encapsulated nucleoside modified mRNA, uridine containing mRNA, and self-amplifying mRNA	BioNTech, fosun pharma, and pfizer	2	0, 28	Phase III NCT04368728
					Phase II 2020-001038-36, ChiCTR2000034825, NCT04537949, NCT04588480
					Phase I NCT04368728
CVnCoV	mRNA	CureVac	2	0, 28	Phase II NCT04515147
					Phase I NCT04449276
LNP-nCoVsaRNA	Self-amplifying mRNA	Imperial college london	2	—	Phase I ISRCTN17072692
ARCT-021	Self-replicating RNA	Arcturus therapeutics, inc. and Duke-NUS	—	—	Phase I/II NCT04480957
Unnamed	mRNA	PLA, walvax biotechnology, and abogen biosciences	2	0, 14 and/or 0, 28	Phase I ChiCTR2000034112 ChiCTR2000039212
ChulaCov19	mRNA	Chulalongkorn university	2	0, 28	Phase I NCT04566276

### Vaccines in Phase III Trials

#### mRNA-1273

The US-based company Moderna, Inc., in partnership with the NIAID, has designed and developed an LNP-formulated mRNA vaccine candidate, named mRNA-1273, the first vaccine to enter clinical trials for immunogenicity and safety assessment (Borah et al., 2020; Poland et al., 2020). The sequence for mRNA-1273 encoding for the pre-fusion stabilized form of S protein was first recognized in mid-January 2020. On administration, the mRNA vaccine prompts cellular production of the antigenic S proteins to initiate a host immune response. After excellent preliminary findings, the Vaccine Research Center of the NIAID started human phase I trials including about 15 subjects (age ranging from 18–55 years) per dose cohort, with three different doses (i.e. 25, 100 and 250 μg) injected intramuscularly at 28 days interval (Jackson et al., 2020). Interim results from published preliminary data showed that there were no serious adverse effects except for one subject who received the first dose of 25 μg and reported transient urticaria, and has been withdrawn afterward. Although administration of the first dose did not induce fever, some subjects in the 100 μg (N = 6, 40%) and 250 μg (N = 8, 57%) groups showed fever-like symptoms. Pain at the injected site was one of the major events reported among the other Grade 1 and Grade 2 adverse events. Apart from these, myalgia, fatigue, headache, and chills were also observed due to systemic or local reactions. Severe systemic events were seen in 21% of subjects receiving 250 μg dose. From the immunogenicity perspectives, dose-dependent specific antibody response was evident, which was at peak on day 15 after the first dose (Jackson et al., 2020). Neutralizing antibodies (S-2P and RBD-specific antibodies) were in a detectable range only in half of the subjects after the first inoculation but were found in all subjects after the second dose. These findings inferred the need for a 2-dose regimen. The CD4^+^ cell-mediated responses were observed in 25 and 100 μg doses with a lower CD8^+^ cell response following the second dose of 100 μg (Jackson et al., 2020). However, it will be interesting to see the data of the second group with older subjects (aged ≥55 years) with altered immunity. On May 11, 2020, Food and Drug Administration (FDA) granted a fast track designation to the mRNA-1273 vaccine (Moderna, Inc., 2020a).

A phase IIa, double-blind, randomized trial of mRNA-1273 including 600 healthy subjects (age ≥18 years) is currently assessing the immunogenicity, safety, and possible adverse reactions of mRNA-1273. In these dose-confirmation study, the participants were divided into total of eight groups depending on the dose and age either receiving vaccine or placebo (NCT04405076). A phase III randomized trial (NCT04470427) incorporating quadruple blinding study was initiated by July 2020 to assess the efficacy of 100 μg dose (highest dose of phase I trial) of the vaccine (Corbett et al., 2020; Jackson et al., 2020). Moderna, Inc. aimed to enroll about 30,000 subjects (age ≥18 years), to be divided into groups receiving either vaccine or placebo guided by broad inclusion criteria, including subjects with stable pre-existing conditions not necessarily requiring therapy changes 3 months before the enrollment. Although there was recruitment of the subjects for the phase III trial, there remain certain concerns related to the lack of ethnic and racial diversity among the subjects, given the disproportionate among the Latino and Black communities (Cohen, 2020). Therefore, it will be interesting to observe the outcomes of the large phase II and III clinical trials. On November 16, 2020, in Moderna’s press release it was reported that the first interim results of the phase III study of mRNA-1273 fulfills its primary efficacy endpoint with an efficacy of 94.5% (Moderna, Inc., 2020b). On December 18, 2020, the U.S. FDA authorized the emergency use of mRNA-1273 among individuals aged 18 years and above. On December 23, 2020, Health Canada authorized the immunization of people ≥18 years of age with mRNA-1273 under an Interim Order. Similarly, on January 4, 2021, Israel’s Ministry of Health authorized the importation of the vaccine in Israel (Moderna, Inc., 2021). On January 6, 2021, European Medicines Agency (EMA) recommended granting a conditional marketing authorization for mRNA-1273 across the European Union (EU) to prevent COVID-19 in subjects above 18 years of age (European Medicines Agency, 2021; Moderna, Inc., 2021). Additional authorizations are currently under review in Switzerland, Singapore, and the United Kingdom (United Kingdom) (Moderna, Inc., 2021). However, data on long-term protection and safety issues of mRNA-1273 is still awaited.

#### BNT162

BioNTech, a German-based company in collaboration with an American company, Pfizer and Fosun Pharma has developed four mRNA-based vaccines (named as BNT162a1, b1, b2, and c2), comprising separate mRNA genes encoding for different antigens (Pfizer, 2020b). Particularly, two of them consist of nucleoside modified mRNA, and the other two consist of uridine containing mRNA and self-amplifying mRNA, respectively. The preliminary data of BNT162b1, an LNP-formulated mRNA vaccine that encodes for the S protein, from 45 participants (age ranging from 18–55 years) divided into either receiving vaccine dose of 10 μg (N = 12), 30 μg (N = 12) and 100 μg (N = 12), or placebo (N = 9) (Mulligan et al., 2020). Subjects receiving 10 and 30 μg doses were given a booster intramuscular dose with an interval of 20 days, whereas the group receiving a 100 μg dose did not require a second dose. Adverse events like headache, fatigue, fever, chills, and myalgia were more frequent in the vaccine group, with fever in 50% of subjects receiving the highest dose in the initial week after immunization. Interim results support the elevated IgG levels peaked at the seventh day after the second dose that lasts for another 14 days. For 100 μg dose, the peak for IgG level was observed at 21st day and did not increase later on. Interestingly, no significant differences in immune responses were observed in the groups receiving 30 and 100 μg following the first dose. These reports argue for the best candidates among 10 and 30 μg doses to proceed into future trials (Mulligan et al., 2020). BNT162b2 was given priority for further development over BNT162b1 because of its desirable immunogenicity and better tolerability profile. However, initial reports from phase I/II trials revealed weaker immune responses in the 65–85 years age category, and most of the enrolled subjects were white and non-Hispanic (Pfizer, 2020a). Importantly, the US government has given funding of USD 1.95 billion to support the large-scale manufacturing of about 100 million doses of their mRNA candidate vaccine (BARDA, 2020). Interestingly, in a recent press release on November 18, 2020, Pfizer announced the successful completion of the efficacy portion of the phase III trial. The interim analysis revealed 95% effectiveness of BNT162b2 in preventing the symptomatic SARS-CoV-2 infection (STAT, 2020). Based on the excellent results obtained in the large trials, on December 2, 2020, BNT162b2 (under the brand name of COMIRNATY) became the first fully-tested vaccine to be approved for emergency use by the United Kingdom regulators (Ledford et al., 2020). Following the positive opinion of EMA’s Committee for Medicinal Products for Human Use (CHMP), on December 21, 2020, the European Commission (EC) has granted a CMA to the vaccine for active immunization to prevent COVID-19 in individuals aged 16 years and above (Pfizer, 2020c). COMIRNATY has now received conditional marketing authorization, emergency use authorization (EUA), or temporary authorization in more than 40 countries across the globe, including all 27 member states of the EU (Businesswire, 2020b). Earlier Pfizer and BioNTech announced an agreement with the EC to supply about 200 million doses of vaccine to the EU member states, with an option of purchasing an additional 100 million doses in 2021 (Pfizer, 2020c).

### Vaccines in Phase I And/Or Phase II Trials

#### CVnCoV

CVnCoV is an mRNA vaccine against COVID-19 being developed by CureVac with support from the German federal government. The vaccine is designed to provide a robust and balanced immune response by using non-chemically modified nucleotides in the mRNA (CureVac, 2020). Data from the pre-clinical experiments in mice and hamsters demonstrated neutralizing titers against the virus, and balanced humoral and cellular immune responses. Currently, the vaccine is in phase I trial involving 168 healthy volunteers in Belgium and Germany (NCT04449276). A preprint of results of the phase I study suggested CVnCoV to be immunogenic, safe, and well tolerated in the subjects (Kremsner et al., 2020). Currently, a multicenter, controlled, phase IIa, the dose-confirmation study is recruiting in Panama and Peru to assess the immunogenicity, safety, and reactogenicity in 691 adults of 18–60 years and above 60 years age (NCT04515147). As per the US Securities and Exchange Commission (SEC) filing, CureVac is planning for a phase III trial with up to 20,000 subjects (COVID-19 Vaccine Tracker, 2020).

#### LNP-nCoVsaRNA

LNP-nCoVsaRNA is a self-amplifying RNA vaccine candidate developed by the Imperial College London within 14 days of first genetic sequencing. LNP-nCoVsaRNA consists of purified synthetic mRNA, which can mimic the viral S protein. Presently, a phase I/II, controlled, randomized trial (COVAC1) is ongoing in 320 healthy volunteers between 18–45 years (for dose-escalation study) and 18–75 years of age (for expanded safety study) (ISRCTN17072692), with a plan for efficacy trial involving about 6,000 subjects. An initiative, called VacEquity Global Health was established by Imperial College London in collaboration with the Morningside Ventures to achieve equity in supply. LNP-nCoVsaRNA is being supported by the United Kingdom Secretary of State for Business, Energy and Industrial Strategy, and the United Kingdom Secretary of State for Health (COVID-19 Vaccine Tracker, 2020).

#### ARCT-021 (Earlier LUNAR-COV19)

Arcturus Therapeutics, Inc. in partnership with Duke-National University of Singapore (NUS) is developing a potential vaccine named ARCT-021, which consists of Arcturus’ self-replicating RNA within a nanoparticle-based formulation capable of inducing CD8^+^ cell-mediated and Th1/Th2-mediated immunity (Alwis et al., 2020). A randomized, placebo-controlled, double-blind, phase I/II study in an estimated 92 healthy volunteers is underway to determine the immunogenicity, safety, tolerability, and ascending dose of ARCT-021 (NCT04480957). The interim results announced by Arcturus revealed an immune response for a single dose as well as prime-boost regimens with well-tolerability. Based on these results, a single vaccine dose of 7.5 µg with prime-boost regimens are being selected for the later stage clinical trials (Biospace, 2020).

#### mRNA Vaccine by PLA/Walvax Biotech/Abogen Biosciences

Another mRNA (unnamed) is being developed and investigated by the Academy of Military Science of the Chinese People's Liberation Army (PLA) in collaboration with Walvax Biotechnology Co., Ltd., Yunnan and Abogen Biosciences Co., Ltd., Suzhou. A phase I trial is being carried out to determine the immunogenicity, safety, and tolerability of three different doses (low, medium, and high dose) of the vaccine in subjects aged 18–60 years and above (ChiCTR2000034112; and ChiCTR2000039212).

### Challenges and Opportunity Gaps in RNA Vaccine Development in Pandemic Situation

Although evidence suggests several advantages of RNA-based vaccines, challenges exist in both research and development and policy-making for determining the precautionary and preparatory stages in the development and immunization against SARS-CoV-2. For instance, recent reports of rare cases of moderate to severe reactions for potential mRNA vaccines have raised concerns over immunogenicity and safety, including the primary findings of the mRNA-1273 phase I trial (Yan et al., 2020). Therefore, it is necessary to understand the possible risks of the RNA vaccine platform, including the local and systemic inflammatory responses, the persistence of induced antigen expression, generation of auto-reactive ensure antibodies and toxic effects associated with delivery components (Pepini et al., 2017; Peck and Lauring, 2018). Even though the S protein is a plausible antigen for immunogenicity, optimized antigen designing is critical in achieving the desired immune response. Moreover, the debate continues over the selection of the best optimization approach, i.e. whether to target the full-length S protein or only the RBD. Similarly, higher glycosylation of the structural proteins assists in the successful viral invasion, and replication inside the host, surviving the host immune responses (Vigerust and Shepherd, 2007; Watanabe et al., 2019). Consequently, glycosylation is a factor, which may reduce the success rate of potential vaccine candidates. Notably, SARS-CoV-2 exhibits an overabundance of glycan sites (Watanabe et al., 2020). The atypical glycosylation observed in SARS-CoV-2 represents quicker mutations, making the vaccine development process extremely difficult. Of importance, the RNA-based platform targeting only the S protein, instead of the entire virus particle, may produce S protein-specific antibodies without being influenced by the viral glycosylation (Wang et al., 2020c). Additionally, precise bioinformatics analysis to determine the involvement of membrane-related co-receptor complex is highly expected to comprehend the rate-limiting conformation of antibodies that affect the ACE2 attachment of virus. The stoichiometric association between the S protein and immune response requires consideration of the intrinsic ratio of the nucleotides per S protein and S protein per SARS-CoV-2. The exact mechanisms of self-defense proteins and the S protein configuration, which affects receptor affinity and viral tropism, are yet to be described. Therefore, further in-depth studies are required to determine the structure and physiological and immunological properties of the structural proteins utilized for the mRNA vaccine development.

On the other hand, it is necessary to ensure adequate intracellular mRNA delivery, ideally by *in vivo* targeting of APCs. Over the past years, there exists a large knowledge gap on the *in vivo* behavior of mRNA, both for naked mRNA and nanoparticle formulations designed for various routes (Verbeke et al., 2019). The current trend of LNP-based formulation must address the overall RNA transfection capacity based on the ability to transport across intra- and extracellular membrane barriers while preventing the potential nanoparticle-induced immune toxicities. Another hard-to-find balance is between the adequate immunogenicity and mRNA-induced antigen expression, which are inherently associated with the structural properties of the mRNA. On the top, approaches like passive immunization with mRNA and replacement of IFN-mediated responses by superior controllable adjuvant systems require further exploitation to justify their benefits over the conventional mRNA vaccines.

Notably, the development of vaccines against Ebola, Zika, and SARS has faced quite different paths. Although the development of the Ebola vaccine was a great success, the disappearance of Zika, and SARS outbreaks before the completion of vaccine development have led to the reallocation of funds from the federal funding agencies, leaving the developers with a financial crisis to set back other vaccine technologies (Fuller and Berglund, 2020). However, the success of oligonucleotide-based therapeutic delivery (Lundin et al., 2015) has opened up new avenues to RNA-based vaccine development, providing several advantages, such as lack of genomic integration of the RNA, lack of antigen persistence, absence of autoantibody production, possible large-scale up, and high purity (Pascolo, 2008). Thus, mRNA-based vaccines present themselves as a promising choice for vaccine development against SARS-CoV-2. Although correlation with immunogenicity may be extrapolated from the past experiences with SARS and MERS vaccines, yet they are not fully understood. The duration of immunity following vaccination is completely unspecified like any naturally acquired infection. Moreover, the ability of a single-dose vaccine to render immunity is uncertain.

Unfortunately, the storage conditions of mRNA vaccines had not been given much attention before the COVID-19 pandemic. Usually, small batches were manufactured and kept at −70°C before administering during initial studies (Pardi et al., 2018). Undoubtedly, the new mRNA based COVID-19 vaccine formulations require stabilization at higher temperatures, as demanded by their storage, transport, and delivery across the world in billions of doses to contain the ongoing pandemic. Available information on the mRNA-1273 profile suggests the vaccine to be stable at −20°C up to 6 months while retaining its stability for about 30 days when kept under refrigeration (Businesswire, 2020a; Crommelin et al., 2021); thus, providing storage and shipping advantage over BNT162b2, which requires an ultra-freeze storage condition (−80 to −60°C) in which it remains stable up to 6 months, while retaining stability up to 5 days under refrigerated conditions (Crommelin et al., 2021; Philippidis, 2020). However, Pfizer and BioNTech claimed to develop temperature-controlled thermal shippers using dry ice to maintain the temperature within −70 to +10°C (Philippidis, 2020). In the earlier literature, the lyophilized RNActive platform was reported to remain active for 6 months and 3 years on storage at 40°C and 5–25°C, respectively (Alberer et al., 2017). Another report suggests that freeze-dried (distilled water or trehalose) naked mRNA remains stable for about 10 months under refrigeration (Jones et al., 2007). Thus, a superior formulation for mRNA delivery is an unmet need of the developmental stage. It is expected that nanoparticle-based formulation could warrant vaccine stability (Probst et al., 2006). Lipid-encapsulated mRNA has shown stability for at least 6 months (Pardi et al., 2018) but long-term storage of such mRNA formulation in an unfrozen form has not yet been reported. Similarly, the LNP-encapsulated mRNA COVID-19 vaccine (ARCoV) manufactured as a liquid formulation can be stored at room temperature (25°C) for at least 1 week (Zhang N. N. et al., 2020). Additionally, incorporation of the RNASE inhibitor within the mRNA vaccine co-formulation and the development of newer purification methods to remove the undesirable reaction components can also be sought as the best ways to improve the platform technology (Probst et al., 2006). Better insights into the novel *in vivo* delivery approach for augmenting mRNA uptake efficiency and plausible effective immune signaling pathways are anticipated soon.

Pre-clinical experiences with potential vaccine candidates against MERS and SARS have raised issues about worsening the lung conditions, either directly or *via* antibody-dependent enhancement (ADE), an adverse event associated with Th2 response. It has been observed that non-RBD-directed antibodies mainly characterize the ADE; thus, it is conceivable that the optimal use of RBDs as vaccine antigens will reduce the risk for ADE. Yet, it remains unclear whether RBD alone will elicit neutralizing antibodies or not (Xu, 2020). Further investigations are still needed to exploit the possible mechanisms for down-regulating the innate immune response to the inoculated mRNA vaccine. Therefore, suitable pre-clinical testing and careful safety monitoring in the clinical trial settings will be critical. Though the ACE2 transgenic mice-an infection model has been established, challenges like tedious process and requirement of expanded facilities are unlikely to be resolved soon. Besides, only a limited number of laboratories have successfully isolated the live strains of SARS-CoV-2. Thus, it is quite difficult to assemble all the desired elements in an animal challenge model. On the other hand, the requirement of a large-animal Biosafety level-3 facility, which provides a challenge for re-access, is a concern with the non-human primate model. Somehow it is still too early to describe a superior animal model as rhesus macaques are quite promising, and so do the ferrets and hamsters (Lurie et al., 2020). Ultimately, consideration of other alternative surrogates, e.g. neutralization *in vitro* assays, could assist in efficient vaccine development. Likewise, the neutralization antibody titer to RBD and the ratio of RBD-directed antibody responses to the full-length S protein can also be considered as suitable surrogates. From a safety perspective, vaccine development must undergo a time-consuming and complicated process, from the early-stage till the marketing of the vaccine. As the traditional vaccine development approaches involving a novel virus target, novel vaccine platforms, and novel development paradigms are likely to be tedious and risky, accelerating the pace in the pandemic era while maintaining the safety concern is still a critical issue to resolve. As mentioned above, the utilization of alternative surrogates for the testing of immunogenicity should be incorporated in the guidelines for vaccine development. Furthermore, approval of the phase 0 trial, if found to be devoid of severe adverse-events in primate immunization, may embolden the innovation of new vaccine development (Xu, 2020). Nevertheless, among the active vaccine candidates, a majority (∼above 70%) are being developed by private industrial sectors, with the remaining being led by the public sector, academic, and other non-profit organizations. Even though few large multinational developers, including Pfizer, Sanofi, Janssen, and GlaxoSmithKline have commissioned vaccine development against COVID-19, most of the lead developers are either meant for small scale manufacturing or are inexpert in large-scale production. Thus, strong international coordination between vaccine production and supply, funding agencies, policy-makers, and the government is of utmost importance to meet the global demand (Lurie et al., 2020).

## Conclusion

Since the first publication on *in vivo* mRNA delivery, RNA has presented itself as a versatile and promising platform for vaccine development. During the COVID-19 pandemic, several biotech companies have started working on the RNA molecule for clinical translation. The RNA-based platform technology prompts rapid refinement with nearly limitless combinations in terms of antigen optimization to a simple and robust manufacturing process. Considering the typical RNA approaches, viz. conventional and self-replicating constructs, many quality attributes to improvise the efficiency and stability of protein expression remain an intense area of development. It is well-known that cytoplasmic mRNA delivery is essential to induce a robust and durable immune response. The self-amplifying and *trans*-amplifying RNA vaccines are capable of providing augmented and prolonged *in vivo* antigen production along with potent intrinsic innate immune-stimulatory functions, and dose-sparing property to meet the demand for a suitable vaccine. Accordingly, significant advances have been made with an emphasis on the novel lipid formulation techniques and the next generation delivery approaches. Overall, progress to date in the RNA engineering, delivery, and construction have represented an RNA platform for further development of a novel vaccine against COVID-19 as the probable first-ever approved RNA vaccine. Although it is conceivable that the majority of the vaccine candidates will fail in the clinical trials, one effective vaccine for the ongoing pandemic would be enough to halt further progress. The preliminary success of mRNA-1273 is a beacon of hope in the present scenario. Given the drastic short time being allocated to the pre-clinical vaccine development, a higher proportion of the potential RNA candidates are expected to fail in the pre-clinical as well as in the clinical stages. On the other hand, if the pandemic disappears abruptly before the vaccines are available for clinical use, developers should continue to stockpile the most promising RNA vaccine candidates and keep them ready for further trials and emergency use authorization as a critical element of future preparedness for similar outbreaks. Nevertheless, the global COVID-19 vaccine development efforts should be guided by ethics, speed of manufacturing, deployment at scale, fairness in allocation, and equity in global supply.

## Abbreviations

ACE: Angiotensin converting enzyme
ARDS: Acute respiratory distress syndrome
COVID-19: Coronavirus disease-2019
DAMPS: Damage-associated molecular patterns
DCs: Dendritic cells
DDA: Dimethyldioctadecylammonium bromide
DNA: Deoxyribose nucleic acid
DOTAP: 1,2-dioleoyl-3-trimethylammonium-propane
dsRNA: Double stranded RNA
EC: European commission
EMA: European medicines agency
EU: European union
HLA: Human leukocyte antigen
IFN: Interferon
IL: Interleukins
IVT: *In vitro* transcribed
LNP: Lipid-derived nanoparticle
MDA5: Melanoma differentiation-associated protein 5
MERS-CoV: Middle east respiratory syndrome
MHC: Major histocompatibility complex
mRNA: Messenger RNA
NIAID: National institute of allergy and infectious diseases
PAMP: Pattern associated molecular patterns
PRPs: Pattern recognition receptors
RBD: Receptor binding-domain
RIG-1: Retinoic acid-inducible Gene 1
RNA: Ribonucleic acid
SARS: Severe acute respiratory syndrome
SARS-CoV-2: Severe acute respiratory syndrome coronavirus 2
TLRs: Toll-like receptors
TNF: Tumor necrosis factor
UTR: Untranslated region
WHO: World health organization
